# The impact of air pollutants on emergency ambulance dispatches due to mental and behavioral disorders in Shenzhen, China

**DOI:** 10.1186/s12889-025-21781-w

**Published:** 2025-02-18

**Authors:** Yu-Chen Tian, Zi-Ming Yin, Peng Wang, Lei Li, Su-Li Huang, Jin-Quan Cheng, Hong-Wei Jiang, Ping Yin

**Affiliations:** 1 Department of Epidemiology and Biostatistics, School of Public Health, Tongji Medical College, Huazhong University of Science and Technology, 13 Hangkong Road, Qiaokou District, Wuhan, 430030 China; 2 Children’s Hospital of Nanjing Medical University, Nanjing, 211112 China; 3 School of Public Health, Shenzhen University, Shenzhen, 518060 China; 4 Shenzhen Center for Disease Control and Prevention, Shenzhen, 518055 China

**Keywords:** Emergency ambulance dispatches, Mental and behavioral disorders, Air pollutants, Interaction effects

## Abstract

**Background:**

The relationships between air pollutants and mental and behavioral disorders (MBDs) remain unclear. We aimed to identify the primary pollutants affecting mental health and evaluate the short-term effects on emergency ambulance dispatches (EADs) due to MBDs.

**Methods:**

Time-stratified case-crossover study and conditional logistic regression model were adopted to explore the impact of air pollutants on EADs due to MBDs from 2013 to 2020 in Shenzhen, China. In order to clarify the influence of gender and age on association, subgroup analysis was carried out. We also applied binary response surface model and distributed lag interaction model to examine the interaction effects between pollutants and meteorological factors on EADs due to MBDs.

**Results:**

Nitrogen dioxide (NO_2_) was the primary pollutant in Shenzhen that affects the EADs due to mental and behavioral disorders, exhibiting significant immediate exposure effects and cumulative lag effects. As NO_2_ concentration increased, the risk of EADs due to mental and behavioral disorders showed a linear upward trend without a threshold. For each interquartile range (IQR) increase of NO_2_, the odds ratio (OR) associated with MBDs was highest at lag 2 in the single-day lag pattern (OR = 1.035, 95% CI: 1.012–1.060) and the effect of NO_2_ reached its maximum at lag 0–6 with OR of 1.078 (95% CI: 1.037–1.122). We did not observe significant associations between PM_2.5_, PM_10_, SO_2_, O_3_ and CO exposures and EADs due to MBDs. In addition, there was an interaction effect between NO_2_ and Humidity index (Humidex). Both high and low Humidex would aggravate the influence of pollutants on mental health.

**Conclusions:**

Short exposure to NO_2_ was positively associated with acute onset of MBDs in Shenzhen, China. Health departments should take effective measures to raise public awareness of NO_2_ and Humidex, as well as their interaction effects.

**Supplementary Information:**

The online version contains supplementary material available at 10.1186/s12889-025-21781-w.

## Background

Mental and behavioral disorders (MBDs) are mental problems with diagnostic significance, characterized by changes in cognition, emotion, and behavior, and may be accompanied by painful experiences and/or functional impairment, including mood disorders, neurotic disorders, schizophrenia, organic mental disorders and so on. The World Health Organization reported that 970 million people around the world were living with a mental disorder [1]. According to the study of global prevalence of MBDs, 29.2% of the respondents had at least once in their life encountered one prevalent MBDs [2]. Another study reported that the worldwide burden of MBDs was responsible for 13.0% of disability-adjusted life years (DALYs) [3]. Therefore, it is essential to identify the potential risk factors of mental and behavioral disorders to reduce the global MBDs burden.

In addition to established risk factors like social environment and genetic factors [4–7], two other important factors that stand out are air pollutants and meteorological conditions, which increase the risk probability of mental illness, as indicated by prior studies [8–11]. In recent years, research on the correlation between pollutants and mental health has predominantly focused on the association between exposure to pollutants and specific mental disorders, often based on hospitalization data, with less attention given to widespread non-pathological diagnoses of mental disorders [12]. Many previous studies have supported the adverse effects of long-term exposure to air pollution on mental health. For instance, a study based on the UK Biobank found an association between long-term exposure to air pollutants and the onset of depression and anxiety [13]. A large-sample cross-sectional study indicated that for every 10 μg/m^3^ increase in PM_2.5_, the risk of adults experiencing symptoms related to nervous tension, depression, and anxiety doubles [14]. Additionally, a multicity case-crossover study conducted in Canada had shown statistically significant associations between exposure to SO_2_, O_3_, and depression [15]. Although multiple studies have explored the relationship between pollutants and mental health, there remains inconsistency and uncertainty regarding which pollutants are the primary factors influencing mental disorders. We aimed to investigate what was the primary pollutant that affected mental health in Shenzhen.

Previously, researchers believed that the impacts of air pollutants and meteorological factors on health was independent. Many aforementioned studies did not consider their interaction effects. In studies examining the relationship between pollutants and mental health, meteorological factors were treated as confounding variables, while in studies investigating the relationship between meteorological factors and mental health, pollutants were treated likewise. However, the health burden resulting from simultaneous exposure to multiple environmental factors may differ from the sum of individual impacts. The mode of action can be synergistic or antagonistic [16]. A meta-analysis examining the modifying effect of temperature levels on the association between pollutants and mortality indicated that high temperatures significantly enhanced the effects of particulate matter and ozone on non-accidental mortality and cardiovascular disease mortality [17]. However, some studies have found that the interaction effects between temperature and pollutants are not statistically significant, and their results are not yet consistent [18]. Regrettably, few studies investigated the interaction effects of air pollutants and meteorological factors on MBDs yet.

In our study, we aimed to study the relationships between air pollutants and emergency ambulance dispatches (EADs) due to MBDs. We employed time-stratified case-crossover study and conditional logistic regression model to explore the primary pollutants that affect mental health in Shenzhen. Despite Shenzhen being a subtropical city with relatively low pollution levels, our research addressed a gap in the literature, which has predominantly focused on cities with higher pollution levels. Moreover, our study employed both a binary response surface model and a distributed lag interaction model to investigate the interaction effect between pollutants and meteorological factors on mental health. We sought to determine how specific weather conditions could exacerbate the impacts of pollutants on mental health. Therefore, the study is of great public health significance to decrease the rate of emergency admission for MBDs and alleviate the burden of mental illness in Shenzhen.

## Materials and methods

### Research area

This research was carried out in Shenzhen, a seaside city in the south of China (Figure S1). At the end of 2020, Shenzhen has an area of 1997.47 km^2^ and a permanent population of 17.63 million. It is located at a low latitude with a subtropical monsoon climate. The climate is mild all year round, with abundant rainfall and sunshine. The air pollution in Shenzhen is characterized by a combination of automobile exhaust and industrial emissions. The number of motor vehicles in the city has been steadily increasing, resulting in a growing contribution of tailpipe emissions to air pollution.

### Data collection

The data, including EADs data, air pollutants data, and meteorological data, were collected from 2013 to 2020 in Shenzhen. Shenzhen First-aid Command Center provided EADs data including the date, time, and cause for the dispatch, the patient’s name, age, gender, residence, symptoms, chief complaints, and initial diagnosis. MBDs were defined according to the 10th Revision of International Classification of Diseases (ICD-10), and their disease codes were F01–F99. Data on air pollutants were sourced from the ChinaHighAirPollutants (CHAP) database provided by the National Earth System Science Data Center [19]. The air pollutants variables included particulate matter less than 2.5 μm in aerodynamic diameter (PM_2.5_, μg/m^3^), particulate matter less than 10 μm in aerodynamic diameter (PM_10_, μg/m^3^), nitrogen dioxide (NO_2_, μg/m^3^), sulfur dioxide (SO_2_, μg/m^3^), ozone (O_3_, μg/m^3^), carbon monoxide (CO, mg/m^3^). The spatial resolution of the PM data set is 1 km×1 km, while the spatial resolution of NO_2_, SO_2_, CO and O_3_ data sets was 10 km×10 km before 2019, and then increased to 1 km [20, 21]. In this study, we geocode the receiving address of each emergency patient using Baidu Maps API (http://lbsyun.baidu.com/). The CHAP data were matched with the location of emergency admission for patients with mental disorders, and individual pollutants exposure was estimated for each patient using bilinear interpolation method, which reduced the bias caused by exposure misclassification. Meanwhile, the daily meteorological data were obtained from the Shenzhen Meteorological Service Center, including the daily mean temperature (Tmean, °C) and relative humidity (RH, %). Considering the combined impact of multiple meteorological factors, this study incorporated Humidity index (Humidex) as a composite meteorological variable into the model to comprehensively capture the impacts of temperature and humidity on mental health. The specific calculation formula for Humidex is detailed in Table S1.

### Study design

In this study, a time-stratified case-crossover design was utilized in conjunction with the conditional logistic regression method to assess the short-term effects between pollutants and EADs due to MBDs in Shenzhen. The day of emergency admission for patients with mental disorders was designated as the case period, while the control periods were selected as the corresponding days of the week within the same year and month as the event day. For example, if January 1, 2013 was chosen as the case period, January 8, 2013, January 15, 2013, January 22, 2013 and January 29, 2013 would be taken as the control period, and so on.

### Statistical analysis

#### Conditional logistic regression model

A conditional logistic regression model was utilized to analyze the relationship between pollutants and EADs due to MBDs across various lag days. Lag modes encompassed single-day lag and cumulative lag. The lag effect of pollutants typically exhibits a brief duration, with a maximum lag period of seven days being considered in our study [22]. The single-day lag ranges from lag 0 (the day of exposure) to lag 7 (a lag of 7 days), while the cumulative lag extends from lag 0–1 (the moving average of the day of exposure and the day before exposure) to lag 0–7 (the moving average from the day of exposure to seven days before exposure). Humidex was included in the model as a confounding variable. The average daily exposure concentration of each pollutant was treated as a continuous variable and included in the respective models to estimate the odds ratios (OR) and 95% Confidence interval (CI) of mental disorders corresponding to each Interquartile range (IQR) increase in pollutant concentration. OR values (95%CI) were calculated by $OR=\exp (\beta \cdot IQR)$, where *β* was the regression coefficient obtained by conditional logistic regression model.

Subgroup analysis was conducted based on gender (male and female) and age (0–14 years old, 15–39 years old, 40–59 years old, and ≥ 60 years old). By comparing the point estimates and standard errors of the odds ratios at different strata, we aimed to investigate potential effect modification among the strata. The calculation formula is as follows:

$$Z=\frac{{{\beta _1} - {\beta _2}}}{{\sqrt {SE_{1}^{2}+SE_{2}^{2}} }}$$

Where *β*_1_ and *β*_2_ are the model regression coefficients of each strata in the subgroup, and *SE*_1_ and *SE*_2_ are the standard errors of regression estimation of each strata. Additionally, in order to assess the robustness of the analysis results, the following sensitivity analyses were carried out in this study: (1) Two-pollutant models were constructed to evaluate the health impacts of air pollutants, and the disparity between the single-pollutant model and the two-pollutant model was assessed using a likelihood ratio test; (2) Excluded the data in 2020 to check the influence of COVID-19 epidemic on the association between pollutants and mental disorders.

#### Interaction analysis

In this section, we first utilized the non-parametric binary response surface model to investigate the impact of the interaction between air pollutants and meteorological factors on mental health. The tensor product smoothing function established the interaction term between pollutants and meteorological factors, constructed a binary response surface model, and obtained a three-dimensional response surface graph of the interaction effect of pollutants and meteorological factors on EADs due to MBDs. Specific modeling strategies were as follows:

$$\begin{array}{l}\:log\left( {\mu {\:_t}} \right) = \alpha \: + Te({X_{1t}}:{X_{2t}}) + ns\left( {time} \right)\\+ \lambda \: \cdot \:holidayt + \gamma \: \cdot \:dowt.\end{array}$$

Where *μ*_*t*_ represents the expected amount of EADs due to MBDs at day *t*; *α* refers to the intercept; *Te*() stands for tensor product smoothing function *ns(time)* is used to control seasonal and long-term trends; *X*_*1t*_ represents the measured value of pollutants exposed at day *t*, *X*_*2t*_ represents the measured value of meteorological factors exposed at day *t*; *holiday*_*t*_ and *dow*_*t*_ are used to control holiday effect and day of the week effect, respectively. *λ* and *γ* are regression coefficients of the corresponding variables.

We also combined the distributed lag model with tensor product smoothing to construct a distributed lag interaction model to analyze the joint lag effect of air pollutants and meteorological factors [23]. The specific model is as follows:

$$\:log\left(\mu\:t\right)={Z}_{t}^{T}\alpha\:+{X}_{1t}^{T}{\beta\:}_{1}+{X}_{2t}^{T}{\beta\:}_{2}+{X}_{It}^{T}\gamma\:$$

$$\begin{array}{l}\: = Z_t^T\alpha \: + \sum \: _{i = 0}^{{L_1}}{x_1}_{,t - i}\beta {\:_{1i}} + \sum \: _{j = 0}^{{L_2}}{x_2}_{,t - j}\beta {\:_{2j}}\\+ \sum \: _{i = 0}^{{L_1}}\sum \: _{j = 0}^{{L_2}}\gamma {\:_{ij}}{x_{1,t - i}}{x_{2,t - i}}\end{array}$$

Where $\:{Z}_{t}=({z}_{1t},{z}_{2t},\dots\:,1)$ represents covariant vector including seasonal and long-term trends, holiday effect, day of the week effect respectively, and a constant 1 corresponding to intercept parameters; Assume that the time *T* is the length of time series, *L*_1_ and *L*_2_ represents the maximum lag days considered for pollutants and meteorological factors respectively; ${X_{1t}}={({x_{1t}}, \ldots,{x_{1,t - {L_1}}})^T}$ denotes the lagged exposure vector of pollutants, ${X_{2t}}={({x_{2t}}, \ldots,{x_{2,t - {L_2}}})^T}$ denotes the lagged exposure vector of meteorological factors, ${X_{It}}={X_{1t}} \otimes {X_{2t}}$ denotes the interaction term, where $\otimes$ is the Kronecker product; *α* refers to the effect of covariate; ${\beta _1}={({\beta _{10}}, \ldots,{\beta _{1{L_1}}})^T}$ represents the coefficient vector of pollutant exposure lagged main effect, ${\beta _2}={({\beta _{20}}, \ldots,{\beta _{2{L_1}}})^T}$ represents the coefficient vector of meteorological factors exposure lagged main effect, $\gamma =vec(\Gamma )={({\gamma _{00}},{\gamma _{01}}, \ldots,{\gamma _{{L_1}}}{\gamma _{{L_2}}})^T}$ indicates the coefficient vector of interaction effect, and Γ is $({L_1}+1) \times ({L_2}+1)$ matrix of interaction effect coefficient, which implies that the lagged effects of the first exposure depend on the level of the second exposure, and vice versa [23]. The main purpose of this part was to estimate the main effect and interaction effect of the distributed lag interaction model. This study also estimated the marginal distributed lag function. The quantity $\:({e}^{{\beta\:}_{1i}+{x}_{2}^{\ast\:}{\sum\:}_{n=0}^{{L}_{2}}{\gamma\:}_{in}}-1)\cdot\:100\%$ represents the percentage change for EADs due to MBDs that is associated with an increase of one unit in pollutant at lag *i* when the meteorological factors is fixed at $x_{2}^{*}$. Similarly, the quantity $({e^{{\beta _{2j}}+x_{1}^{*}\sum\nolimits_{{m=0}}^{{{L_1}}} {{\gamma _{mj}}} }} - 1) \cdot 100\%$ represents the percentage change for EADs due to MBDs that is associated with an increase of one unit in meteorological factors at lag *j* when the pollutant is fixed at $x_{1}^{*}$.

All statistical analyses were conducted with SAS (version 9.4, Sai Shi Software, Cary, NC, USA) and R software (version 4.3.2, R Foundation for Statistical Computing, Vienna, Austria). The “season”, “mgcv”, and “splines” packages were used to establish the models.

## Results

### Descriptive analysis

Table 1 illustrates the descriptive statistical results for air pollutants, meteorological variables, and EADs due to MBDs during the study period from 2013 to 2020. The mean daily concentration of PM_2.5_, PM_10_, NO_2_, SO_2_, O_3_, and CO were 28.74 μg/m^3^, 46.87 μg/m^3^, 34.45 μg/m^3^, 8.24 μg/m^3^, 71.40 μg/m^3^, and 0.83mg/m^3^, respectively. The average for daily mean temperature, relative humidity, and Humidex were 23.56 °C, 75.72%, and 30.93, respectively. There were 24,967 EADs due to MBDs in total, with an average of 8 cases and a maximum of 27 cases per day. Approximately 51.20% of EADs due to MBDs cases were men, and 43.35% were women. The 15–39 and 40–59 age groups had the largest proportions of EADs due to MBDs (62.45% and 24.76%, respectively) in comparison to the 0–14 and 60 + age groups (1.44% and 5.70%, respectively). There were 1412 cases without age information and 1360 cases without sex information. Figure S2 shows the Spearman correlations between air pollutants and meteorological variables. A strong positive connection existed between the daily concentrations of NO_2_ and the daily concentrations of PM_2.5_, PM_10_, SO_2_, and CO (Spearman correlation coefficients *r* > 0.6, *P* < 0.001). Figure S3 displays the time-series distributions of air pollutants, meteorological factors, and daily EADs due to MBDs from 2013 to 2020. The figure illustrates that pollutants, with the exception of O_3_, generally exhibited a decreasing trend on an annual basis. In contrast, meteorological factors displayed clear periodic and seasonal variations, while the daily EADs due to MBDs generally demonstrated a fluctuating upward trajectory over time.

**Table 1:** Summary of air pollutants, meteorological factors, and EADs due to MBDs counts in Shenzhen, 2013–2020

	Sum(%)	Mean ± SD	Min	*P*_1_	*P*_10_	*P*_50_	*P*_90_	*P*_99_	Max	IQR
Daily air pollutants										
PM_2.5_, μg/m^3^	–	28.74 ± 17.91	3.13	6.09	10.36	24.91	51.18	91.27	137.07	22.08
PM_10_, μg/m^3^	–	46.87 ± 25.85	5.55	12.91	20.14	40.69	80.35	129.88	181.76	32.52
NO_2_, μg/m^3^	–	34.45 ± 15.93	6.73	11.64	18.09	31.43	54.81	89.26	133.71	17.42
SO_2_, μg/m^3^	–	8.24 ± 3.78	3.09	3.73	4.91	7.36	12.27	23.07	54.81	3.62
O_3_, μg/m^3^	–	71.40 ± 36.36	15.36	21.91	33.96	62.80	122.09	193.00	246.36	45.70
CO, mg/m^3^	–	0.83 ± 0.28	0.40	0.44	0.53	0.76	1.24	1.59	1.93	0.38
Meteorological factors										
Tmean, °C	–	23.56 ± 5.31	3.50	10.20	15.90	24.70	29.60	30.80	33.00	8.50
RH, %	–	75.72 ± 12.88	19.00	34.00	58.00	78.00	90.00	97.00	100.00	14.00
Humidex	–	30.93 ± 9.66	0.69	8.84	16.90	32.17	41.93	44.18	46.79	16.27
No. of EADs due to MBDs										
Total EADs due to MBDs ^a^	24,967(100.00)	8.54 ± 4.36	0	1	4	8	14	21	27	–
Gender										
Male	12,784(51.20)	4.38 ± 2.63	0	0	1	4	8	12	16	–
Female	10,823(43.35)	3.70 ± 2.31	0	0	1	3	7	11	16	–
Age										
0–14 years old	359(1.44)	0.12 ± 0.37	0	0	0	0	1	2	3	–
15–39 years old	15,592(62.45)	5.34 ± 2.83	0	0	2	5	9	13	19	–
40–59 years old	6182(24.76)	2.12 ± 1.67	0	0	0	2	4	7	10	–
60 ∼ years old	1422(5.70)	0.49 ± 0.74	0	0	0	0	1	3	5	–

Abbreviations: PM_2.5_, particulate matter less than 2.5 mm in aerodynamic diameter; PM_10_, particulate matter less than 10 mm in aerodynamic diameter; NO_2_, nitrogen dioxide; SO_2_, sulfur dioxide; O_3_, ozone; CO, carbon monoxide; Tmean, daily mean temperature; RH, relative humidity; Humidex, Humidity index; Sum, total number; EADs due to MBDs, emergency ambulance dispatches due to mental and behavioral disorders; SD, standard deviation; Min, minimum; Max, maximum; P, percentile; IQR, Interquartile range

^a^ There are 1360 cases of EADs due to MBDs that lacked gender information and 1412 cases lacked age information

### Associations between air pollutants and EADs due to MBDs

**Fig. 1:**
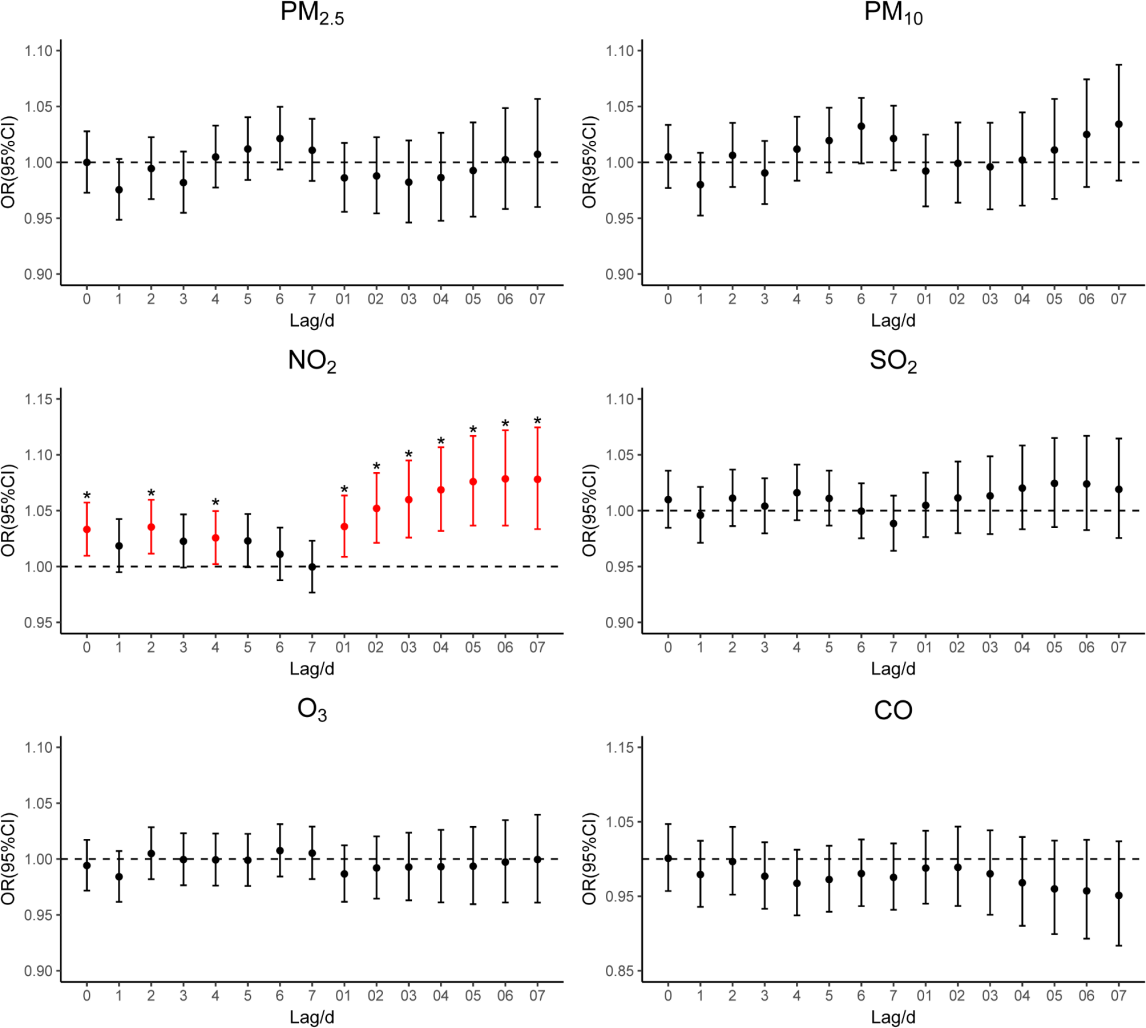
Lag-response curves for six kinds of air pollutants for each IQR increment associated with EADs due to MBDs Abbreviations: PM_2.5_, particulate matter less than 2.5 μm in aerodynamic diameter; PM_10_, particulate matter less than 10 μm in aerodynamic diameter; NO_2_, nitrogen dioxide; SO_2_, sulfur dioxide; O_3_, ozone; CO, carbon monoxide; EADs due to MBDs, emergency ambulance dispatches due to mental and behavioral disorders; IQR, Interquartile Range; * means *P* < 0.05.

**Fig. 2:**
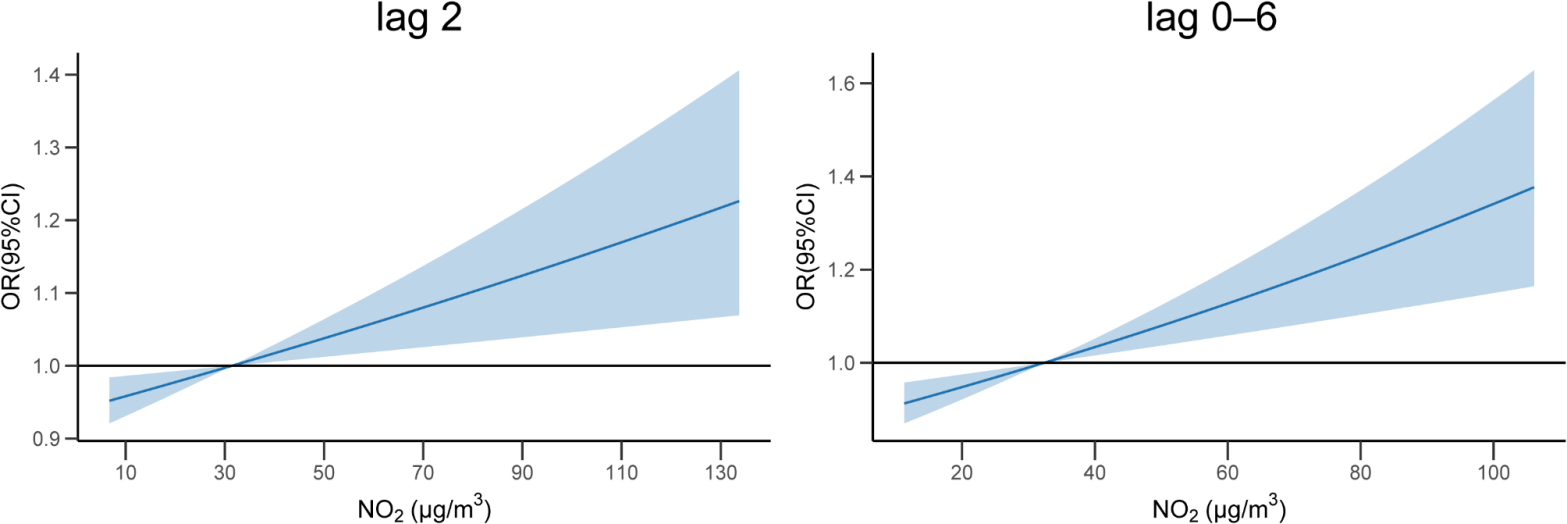
Exposure-response curves between NO2 exposure and EADs due to MBDs when at lag 2 and lag 0–6 Abbreviations: EADs due to MBDs, emergency ambulance dispatches due to mental and behavioral disorders; NO_2_, nitrogen dioxide.

Figure 1 presents the lag-response curves depicting the impacts of six kinds of pollutants on EADs due to MBDs when each increased by an IQR. The results indicated that among the six pollutants examined, only NO_2_ had an immediate exposure effect and cumulative lag effect on EADs due to MBDs. In the single-day lag mode, exposure on the day of exposure (lag 0), two days before exposure (lag 2), and four days before exposure (lag 4) had an effect on EADs due to MBDs. In the cumulative lag mode, from the cumulative lag of 1 day (lag 0–1) to the cumulative lag of 7 days (lag 0–7), NO_2_ had an impact on EADs due to MBDs. There was no significant effect of PM_2.5_, PM_10_, SO_2_, O_3_ and CO exposures observed from Fig. 1. Table 2 shows the specific OR (95%CI) of the impacts of air pollutants on EADs due to MBDs under different lag modes. The highest OR value was observed at lag 2 (OR = 1.035, 95% CI: 1.012–1.060), indicating that with each IQR increase in NO_2_ two days before exposure, the EADs due to MBDs would increase by 3.5%. In the cumulative lag mode, the impact of NO_2_ reached the maximum at lag 0–6, with the OR value being 1.078 (95% CI: 1.037–1.122). This implied that the EADs due to MBDs would increase by 7.8% when NO_2_ daily average concentration increased by an IQR at lag 0–6. Figure 2 shows the exposure-response curves between NO_2_ and EADs due to MBDs when at lag 2 and lag 0–6. The analysis revealed no significant non-linear trend between short-term NO_2_ exposure and EADs due to MBDs. In addition, as the concentration of NO_2_ increased, the risk of increasing the EADs due to MBDs also showed a rising trend and there was no threshold (Table S2).

**Table 2:** Specific odds ratio (95%CI) for EADs due to MBDs associated with each IQR increment in air pollutants concentration under different lag modes

Lag days	Odds ratio (95%CI)					
	PM_2.5_	PM_10_	NO_2_	SO_2_	O_3_	CO
lag 0	1.000(0.973,1.028)	1.005(0.977,1.034)	**1.033(1.010**,**1.057)**	1.010(0.985,1.036)	0.994(0.972–1.017)	1.001(0.957,1.047)
lag 1	0.975(0.949,1.003)	0.980(0.952,1.009)	1.018(0.995,1.043)	0.996(0.971,1.021)	0.984(0.962–1.007)	0.979(0.936,1.025)
lag 2	0.994(0.967,1.023)	1.006(0.978,1.035)	**1.035(1.012**,**1.060)**	1.011(0.986,1.037)	1.005(0.982–1.028)	0.997(0.952,1.043)
lag 3	0.982(0.955,1.010)	0.990(0.963,1.019)	1.023(0.999,1.047)	1.004(0.980,1.029)	1.000(0.976–1.023)	0.977(0.933,1.023)
lag 4	1.005(0.977,1.033)	1.012(0.984,1.041)	**1.026(1.002**,**1.050)**	1.016(0.991,1.041)	0.999(0.976–1.023)	0.967(0.924,1.012)
lag 5	1.012(0.984,1.040)	1.019(0.991,1.049)	1.023(0.999,1.047)	1.011(0.987,1.036)	0.999(0.976–1.023)	0.972(0.929,1.018)
lag 6	1.021(0.994,1.050)	1.032(0.999,1.062)	1.011(0.988,1.035)	1.000(0.975,1.025)	1.008(0.984–1.031)	0.980(0.937,1.026)
lag 7	1.011(0.983,1.039)	1.021(0.993,1.051)	1.000(0.977,1.023)	0.988(0.964,1.013)	1.005(0.982–1.029)	0.975(0.932,1.021)
lag 0–1	0.986(0.956,1.017)	0.992(0.961,1.025)	**1.036(1.009**,**1.064)**	1.005(0.976,1.034)	0.987(0.962–1.012)	0.988(0.940,1.038)
lag 0–2	0.988(0.954,1.022)	0.999(0.964,1.036)	**1.052(1.021**,**1.084)**	1.011(0.980,1.044)	0.992(0.965–1.020)	0.989(0.937,1.044)
lag 0–3	0.982(0.946,1.020)	0.996(0.958,1.035)	**1.060(1.026**,**1.095)**	1.013(0.979,1.049)	0.993(0.963–1.024)	0.980(0.925,1.038)
lag 0–4	0.986(0.948,1.026)	1.002(0.961,1.045)	**1.069(1.032**,**1.107)**	1.020(0.983,1.058)	0.993(0.961–1.026)	0.968(0.910,1.030)
lag 0–5	0.993(0.951,1.036)	1.011(0.967,1.057)	**1.076(1.037**,**1.117)**	1.024(0.985,1.065)	0.994(0.960–1.029)	0.960(0.899,1.025)
lag 0–6	1.002(0.958,1.049)	1.025(0.978,1.074)	**1.078(1.037**,**1.122)**	1.024(0.983,1.067)	0.997(0.961–1.035)	0.957(0.893,1.026)
lag 0–7	1.007(0.960,1.057)	1.034(0.984,1.087)	**1.078(1.034**,**1.125)**	1.019(0.976,1.065)	1.000(0.961–1.040)	0.951(0.884,1.024)

Abbreviations: EADs due to MBDs, emergency ambulance dispatches due to mental and behavioral disorders; IQR, Interquartile Range; PM_2.5_, particulate matter less than 2.5 μm in aerodynamic diameter; PM_10_, particulate matter less than 10 μm in aerodynamic diameter; NO_2_, nitrogen dioxide; SO_2_, sulfur dioxide; O_3_, ozone; CO, carbon monoxide; Statistical significance is shown in bold

The results of subgroup analysis are shown in Table 3 and Figure S4 and the lag mode is lag 0–6. The results indicated that a positive linear correlation between daily NO2 concentrations and EADs due to MBD in subgroup populations. The OR value between NO_2_ and EADs due to MBDs in male population was 1.072 (95% CI: 1.015–1.133), while the OR value between NO_2_ and EADs due to MBDs in female population was 1.060 (95% CI: 0.999–1.125). The *P* value for effect modification exceeded 0.05, indicating that the difference was not statistically significant and gender did not have a modifying effect on the impact of pollutants on EADs due to MBDs. When considering age subgroups, with the 15–39 age group (OR = 1.065, 95% CI: 1.013–1.118) as the reference group, all effect modification *P* values were greater than 0.05, implying that the OR values across different strata were similar. Consequently, there was no significant effect modification between NO_2_ and EADs due to MBDs in age subgroups.

**Table 3:** Specific odds ratios (95%CI) for EADs due to all MBDs and its subgroups associated with an IQR increment in NO2 concentration at lag 0–6

Variables	Odds ratio (95%CI)	Z value	*P* value
Gender		0.270	0.787
Male	**1.072 (1.015**,**1.133)**		
Female	1.060 (0.999,1.125)		
Age			
0–14 years old	1.100 (0.756,1.601)	0.170	0.865
15–39 years old	**1.065 (1.013**,**1.118)**	Ref	
40–59 years old	1.061 (0.980,1.149)	0.066	0.948
60 ∼ years old	1.125 (0.944,1.342)	0.597	0.550

Abbreviations: EADs due to MBDs, emergency ambulance dispatches due to mental and behavioral disorders; NO_2_, nitrogen dioxide; IQR, Interquartile Range; Statistical significance is shown in bold

The sensitivity analysis results are presented in Tables S1 and Figure S5. Based on the NO_2_ single pollutant model, sensitivity analysis was made for other pollutants (PM_2.5_, PM_10_, SO_2_, O_3_, and CO) to construct two-pollutant models. After adjusting the variables in all the two-pollutant models, the relationship between NO_2_ exposure and EADs due to MBDs remained significant, indicating that the model results of NO_2_ single pollutant are robust. The model likelihood ratio test results revealed that the *P* values for the NO_2_ + PM_10,_ NO_2_ + SO_2_, and NO_2_ + O_3_ models were greater than 0.05, suggesting that the fit of these models was not as strong as that of the NO_2_ single pollutant model. Conversely, the *P* values for the NO_2_ + PM_2.5_ and NO_2_ + CO models were less than 0.05, indicating statistically significant differences and the superiority of these models over the NO_2_ single pollutant model. Considering the strong correlation between NO_2_, PM_10_, and CO, the simple NO_2_ single pollutant model was employed to analyze the results. Additionally, after excluding the data post the COVID-19 epidemic, no significant changes were observed in our results (Figure S5). The aforementioned sensitivity analysis demonstrated the stability of our results.

### Interaction analysis results

**Fig. 3:**
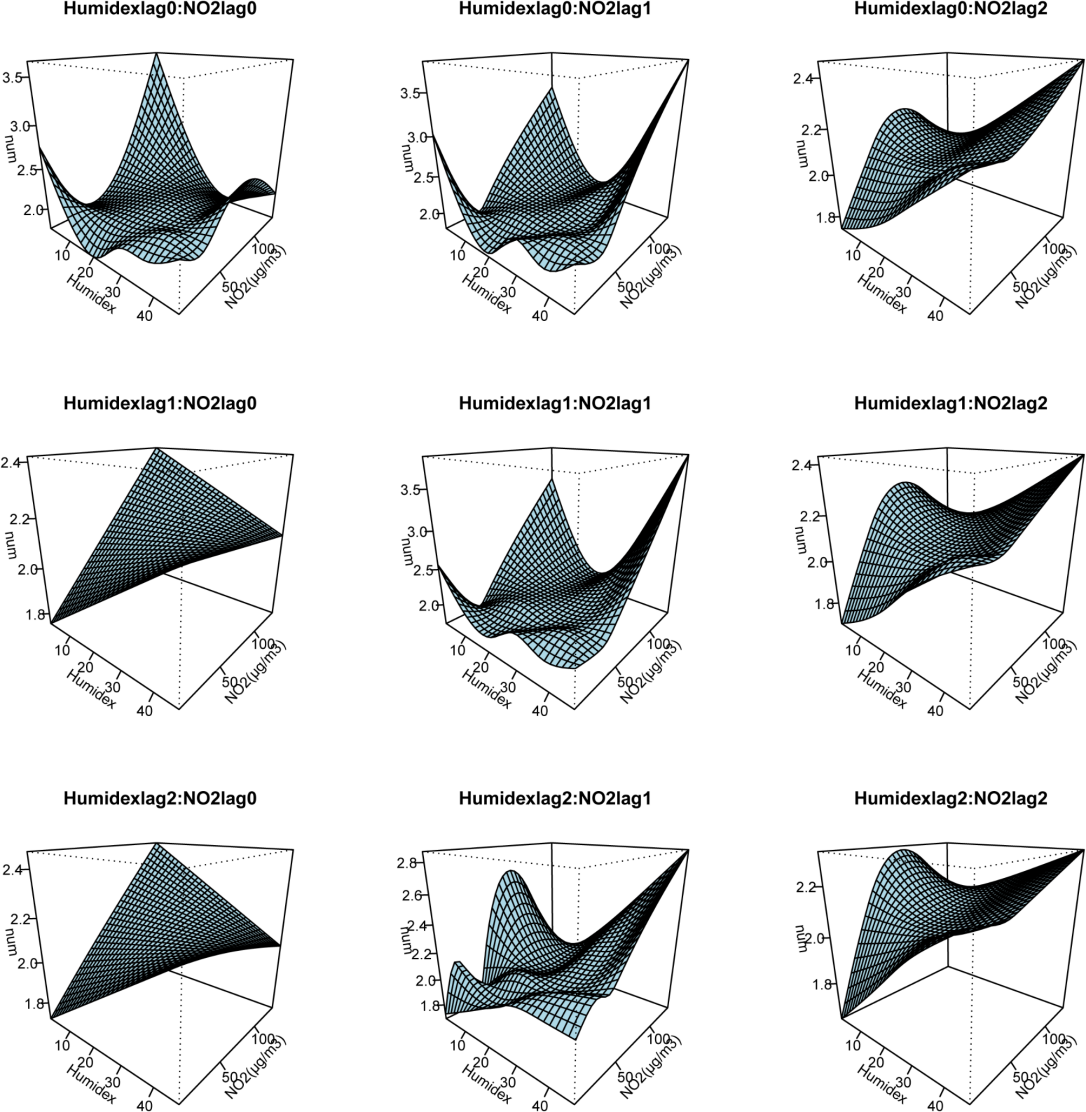
Three-dimensional curved surface diagram illustrating the influence of the interaction between NO2 and Humidex on EADs due to MBDs (lag 0–lag 2) Abbreviations: EADs due to MBDs, emergency ambulance dispatches due to mental and behavioral disorders; NO_2_, nitrogen dioxide; Humidex, Humidity index.

In light of the research presented above, which identified NO_2_ as the primary pollutant affecting the mental health in Shenzhen, this part primarily focused on analyzing the interaction effect of NO_2_ and Humidex on EADs due to MBDs. Due to the short-term lag effect of NO_2_ and Humidex, we first selected a lag of 0–2 days (lag 0, lag 1 and lag 2) to assess their interaction effect by binary response surface model. Figure 3 presents a three-dimensional curved surface diagram illustrating the influence of the interaction between NO_2_ and Humidex on EADs due to MBDs according to binary response model. The Humidex lag 0:NO_2_ lag 0, Humidex lag 0:NO_2_ lag 1, and Humidex lag 1:NO_2_ lag 1 revealed that exposure to low Humidex on the current day and a one-day lag result in an interaction effect with low NO_2_, significantly increasing the risk of EADs due to MBDs. All interaction terms indicated a clear interaction between low Humidex and high NO_2_, with a notable rise in the amount of EADs due to MBDs as NO_2_ concentration increased. Notably, Humidex lag 0:NO_2_ lag 2, Humidex lag 1:NO_2_ lag 2, and Humidex lag 2:NO_2_ lag 2 indicated that low NO_2_ at two-day lag enhances the impact of high Humidex on EADs due to MBDs. The interaction effect between high Humidex and high NO_2_ was particularly obvious. Apart from a slight impact on EADs due to MBDs on the day of exposure, significant increase was observed in other interaction terms. In conclusion, the interaction effect between NO_2_ and Humidex exists and affects the EADs due to MBDs.

**Fig. 4:**
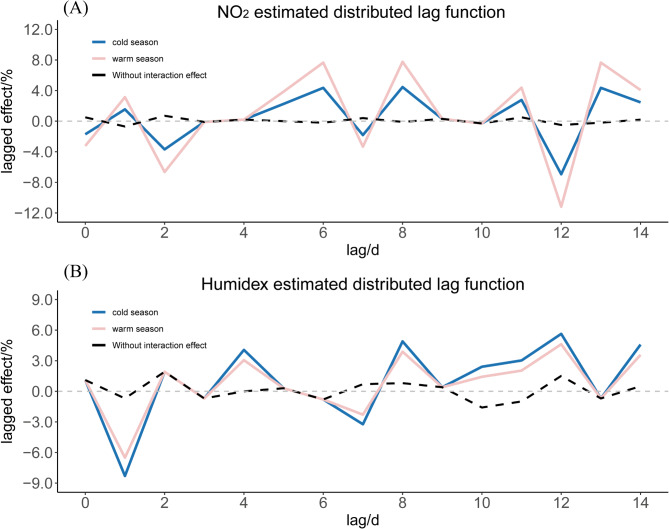
Marginal distributed lag function for the effects of NO_2_ or Humidex on EADs due to MBDs Abbreviations: EADs due to MBDs, emergency ambulance dispatches due to mental and behavioral disorders; NO_2_, nitrogen dioxide; Humidex, Humidity index.

By employing the distributed lag interaction model, this study further investigated the impact of the interaction between NO_2_ and Humidex on EADs due to MBDs. The maximum lag days of NO_2_ and Humidex were set to 14 days. Table 4 shows the relative risk values (RR) of these 225 kinds of interaction terms obtained by the distributed lag interaction model. From the table, it could be observed that the results of the distributed lag interaction model aligned with the findings of binary response surface model in early lag periods. The RR values of the interaction terms on the day of exposure and one-day lag were statistically significant. Table S3 displays the RR values(95%CI) and corresponding *P* values that are statistically significant. On the day of exposure, the RR value of the interaction term between NO_2_ and Humidex was 1.001 (95% CI: 1.000–1.001), while at lag 1, the RR value was 1.002 (95% CI: 1.001–1.003). In terms of late lag periods, Humidex would interact with the long-term lag of NO_2_. For instance, the RR value for the interaction effect between NO_2_ at lag 13 and Humidex at lag 3 was 1.001 (95% CI: 1.000–1.003), and the RR value for the interaction effect between NO_2_ at lag 13 and Humidex at lag 8 was 1.002 (95% CI: 1.000–1.003). This shows that the interaction between NO_2_ and Humidex will have an impact on EADs due to MBDs on a specific lag day. We presented the marginal distributed lag function of NO_2_ and Humidex by integrating out the other variable in Fig. 4. The $x_{1}^{*}$ was the average value of Humidex in cold season and warm season respectively, and $x_{2}^{*}$ was the average value of NO_2_ in cold season and warm season respectively. Figure 4(A) shows the percentage change in EADs due to MBDs that is associated with an increase of IQR in NO_2_ level at different lag days when the Humidex level is at $x_{1}^{*}$. Similarly, Fig. 4(B) represents that the percentage change in EADs due to MBDs that is associated with an increase of IQR in Humidex level at different lag days when the NO_2_ level is at $x_{2}^{*}$. As can be seen from the Fig. 4, when NO_2_ or Humidex changed, its corresponding marginal lag function would also change, indicating that there was a synergistic interaction between NO_2_ and Humidex. We added a dotted curve in each panel for the estimated marginal lag function from a single-variable analysis (i.e. models with NO_2_ alone or Humidex alone), representing the average distributed lag effects if we disregard the interaction effect between NO_2_ and Humidex. From the results in the figure, we can see that the evidence supporting the existence of synergistic interaction effect is compelling.

**Table 4:** Specific odds ratios of 225 kinds of interaction terms obtained by the distributed lag interaction model

NO_2_\Humidex	lag 0	lag 1	lag 2	lag 3	lag 4	lag 5	lag 6	lag 7	lag 8	lag 9	lag 10	lag 11	lag 12	lag 13	lag 14
lag 0	1.001*	0.999	1.000	1.000	1.000	1.000	1.000	1.000	1.000	1.000	1.000	1.000	1.000	1.000	1.000
lag 1	0.999	1.002*	0.999	1.000	1.000	1.000	1.000	0.999	1.001	0.999	1.000	1.000	1.000	0.999	1.000
lag 2	1.000	1.000	1.000	1.001	0.999	1.000	1.001	1.000	1.000	1.000	1.000	1.000	0.999	1.000	1.000
lag 3	1.000	1.000	1.000	0.999	1.001	1.000	0.999	1.001	1.000	1.000	1.000	1.000	0.999	1.001	1.000
lag 4	1.000	1.000	1.000	1.001	0.999	1.001	1.000	1.000	1.000	1.000	1.000	1.000	1.000	1.000	1.000
lag 5	1.000	1.000	1.001	0.999	1.000	1.000	1.000	1.000	1.000	1.000	1.000	1.000	1.000	1.000	1.000
lag 6	1.000	1.000	1.000	1.000	1.000	1.001	0.999	1.000	1.000	1.000	1.000	1.000	1.000	1.000	1.000
lag 7	1.000	0.999	0.999	1.001	0.999	1.001	1.000	1.000	1.000	1.000	1.000	1.000	1.000	1.000	1.000
lag 8	1.000	1.001	1.000	0.999	1.001	0.999	1.001	0.999	1.000	1.001	0.999	1.001	1.000	1.000	1.000
lag 9	1.000	1.000	0.999	1.001*	1.000	0.999	1.001	1.000	1.000	1.000	1.000	1.000	1.000	1.000	1.000
lag 10	1.000	1.000	1.001	0.999	1.000	1.000	1.000	1.000	1.000	1.001	1.000	1.000	0.999	1.001	1.000
lag 11	1.000	1.000	0.999	1.001	1.000	1.000	1.001	0.999	1.001	0.999	1.000	1.000	1.001	0.999	1.001
lag 12	1.000	0.999	1.001*	0.999	1.001	1.000	0.999	1.001*	0.999	1.001	1.000	1.000	1.000	1.001	1.000
lag 13	0.999	1.001*	0.999	1.001*	0.999	1.000	1.001	0.999	1.002*	0.998	1.001	1.000	1.000	1.000	1.000
lag 14	1.000	1.000	1.000	0.999	1.001	1.000	1.000	1.001	0.999	1.001*	1.000	1.000	0.999	1.001	1.000

Abbreviations: EADs due to MBDs, emergency ambulance dispatches due to mental and behavioral disorders; NO_2_, nitrogen dioxide; Humidex, Humidity index; * represents statistical significance for the interaction effect in the distributed lag interaction model at the 5% level (*P* < 0.05)

## Discussion

This is the first study that studied the relationship between air pollutants and mental health in Shenzhen, China. We found that exposure to NO_2_ was significantly associated with increases in EADs due to MBDs. As the concentration of NO_2_ increased, the risk of EADs due to MBDs demonstrated a linear upward trend, with no identifiable threshold. In this study, it was determined that gender and age had no significant effect on modifying the relationship between NO_2_ and EADs due to MBDs, but the OR values of male population and the age group of 15–39 years old were relatively higher. Furthermore, we also explored the influence of the interaction effect between NO_2_ and Humidex on EADs due to MBDs. The results demonstrated that an interaction effect existed between NO_2_ and Humidex at specific lag days and exposure levels, which would increase EADs due to MBDs.

In recent years, research on the relationship between pollutants and mental health has mostly focused on the relationship between air pollutant exposure and a specific mental illness. The research data is also based on hospitalization data, and there have been few studies on the extensive non-pathological diagnosis of mental disorders in the general population [12]. Many previous studies supported the adverse effects of air pollutant exposure on mental health. For example, a study based on UK Biobank found that there was a correlation between long-term exposure to air pollutants and the onset of depression and anxiety [13]. The results of our study showed that among the six types of air pollutants in Shenzhen, only NO_2_ showed immediate exposure effect and cumulative lag effect on mental health. Most studies indicated that NO_2_ exposure would increase the risk of depression, with a certain lag effect, which was consistent with the results of this study [24, 25]. There was no significant effect of short-term exposure to PM_2.5_, PM_10_, SO_2_, CO and O_3_ on EADs due to MBDs in Shenzhen, which is inconsistent with some previous studies from other regions. For example, a large sample study showed that for every 10 μg/m^3^ increase in PM_2.5_, the risk of nervous tension, depression and anxiety-related symptoms in adults increased by 2.31 times [14]. In a study on the relationship between PM_10_ and mental health in Germany, researchers found a certain degree of correlation between mental health and PM_10_ [26]. Some studies have also found a statistical significance between exposure to SO_2_ and O_3_ and depression, with SO_2_ showing a certain lag effect [15]. Many cohort studies have investigated the correlation between air pollutants and mental health, all confirming the detrimental effects of pollutants. However, there remain inconsistencies and uncertainties regarding which pollutants are the primary contributors to mental disorders. This is due to varying levels of social development and the diverse types and severities of pollutants in different countries and regions. Therefore, the relevant research conclusions may not necessarily apply to Shenzhen, and the results of our study should also be cautiously extrapolated. Additionally, there exists a discrepancy between the measurement methods of pollutants based on fixed monitoring stations and model simulations in accurately reflecting the true impact of pollutant exposure. Monitoring station measurements may not accurately depict individual exposure levels, leading to bias in assessing the real effect of pollutants in various studies. To comprehensively understand the relationship between pollutant exposure and mental health, research on pollutants’ influence on mental disorders should focus on individual pollutant exposure.

Our study revealed that NO_2_ exposure at early lag had an impact on EADs due to MBDs. The strongest correlation between NO_2_ and EADs due to MBDs was observed at lag 2. Previous studies have also highlighted the significance of lag time in affecting the relationship between air pollutants and mental health outcomes, and the peak lag effect of air pollutants occurs within 3 days [27]. As NO_2_ concentration rises, the risk of EADs due to MBDs shows a linear upward trend without threshold, which is also consistent with the conclusion that relationship between air pollutants and health outcomes shows a linear trend in previous studies. Our study found no significant effect modification between gender and age in relation to NO_2_ exposure and EADs due to MBDs. However, the OR values for male population and the 15–39 age group were relatively higher, suggesting potential sensitivity to NO_2_ exposure in mental health outcomes. Previous research has found longer lag risk durations for women exposed to pollutants compared to men and the elder, with higher OR value observed in women and young people [15, 28]. This is probably related to the differences in demographic and exposure patterns between study area. As of 2020, Shenzhen had a permanent population of approximately 17.8 million of which only 4.32% are elderly people over 60. Compared with the elderly, young people have more commuting time and outdoor activities, resulting in more exposure to pollutants. Outdoor jobs, such as transportation and construction, are also occupied by young men mostly. The results suggest that young people and men should also pay more attention to protecting themselves by using effective masks when the concentration of pollutant is high.

In the past, researchers believed that the effects of air pollutants and meteorological factors on health were independent. Many studies mentioned above did not consider the interaction between them. When studying the relationship between pollutants and mental health, meteorological factors were regarded as confounding variables, whereas when studying the relationship between meteorological factors and mental health, pollutants were also considered as confounding variables. However, the health burden caused by simultaneous exposure to multiple environmental factors may differ from the sum of individual effects, with possible synergistic or antagonistic modes of action [29]. A meta-analysis to study the modification effect of temperature on the mortality of pollutants pointed out that temperature had statistically significant interaction effect with PM_10_ and O_3_ on unintentional mortality and cardiovascular disease mortality, and high temperature significantly enhanced the effects of PM_10_ and O_3_ on unintentional mortality and cardiovascular disease mortality [17]. However, some studies have found that the interaction between temperature and pollutants is not statistically significant [18]. Therefore, the research results are not yet unified.

One study conducted in Athens was the first to explore the interaction between temperature and air pollution on health using multiple linear regression models. The study concluded that high temperature and various air pollutants have synergistic effects on health [30]. Some studies have utilized hierarchical analysis method based on generalized additive model [31–33]. Additionally, Roberts employed the binary response surface model to explore the interaction effect [34]. These interaction research methods have their own advantages and disadvantages. The binary response surface model can directly observe whether the interaction effect exists, but it can’t quantitatively evaluate its effect. Hierarchical analysis method can be used for quantitative analysis, but the choice of demarcation point is subjective and there is no good stratification standard. So how to accurately conduct model is the key to analyze the interaction effect. Considering the collective impact of multiple meteorological factors, we selected Humidex as the research index of meteorological factors. Humidex, proposed by Masterton and Richardson, is a composite index of temperature and humidity that is utilized to represent individuals’ perceived heat. This index is commonly employed by Canadian meteorologists to convey individual thermal comfort during hot or humid conditions [35]. Compared with the original meteorological indicators, Humidex captures the comprehensive effects of temperature and humidity on mental health more comprehensively. Compared with other meteorological comprehensive indexes, it is a thermal comfort index especially suitable for describing hot and humid weather. Therefore, our study mainly studied the interaction effect between NO_2_ and Humidex. The binary response surface model and distributed lag interaction model constructed in our study proved that the interaction between NO_2_ and Humidex would have an impact on EADs due to MBDs. The synergistic interaction effect between NO_2_ and Humidex increased the risk of EADs due to MBDs under certain lag durations and exposure levels, especially when both NO_2_ and Humidex reached extreme values.

NO_2_ in the atmosphere primarily originates from traffic and industrial emissions, which can be involved in triggering oxidative stress, inflammatory reactions, and neurotoxicity, thereby impacting human mental health. NO_2_ has been implicated in the induction of oxidative stress reactions, leading to the production of oxidizing substances such as superoxide anions and hydroxyl radicals. These oxidizing substances have the potential to disrupt the structure and function of cell membranes, impact neurotransmitter release and receptor activity, and consequently influence mental health [36]. It has been noted that NO_2_ can instigate inflammatory responses and stimulate the production of inflammatory cells and mediators within the body. These inflammatory reactions may compromise normal brain function, resulting in mental health issues such as emotional instability, depression, and anxiety [37]. Furthermore, NO_2_ has the ability to directly impact the nervous system and impair the structure and function of nerve cells. This neurotoxicity can lead to disturbances in neurotransmitter function and neuronal apoptosis, thus affecting mental states and behaviors [38]. In summary, the evidence supporting the influence of NO_2_ on mental health is sufficient and clear from a mechanistic standpoint.

This study builds on previous literature which explored the influence of pollutants on mental health. There are some strengths in our research. First, EADs data may be more sensitive to temperature than hospitalization data. The EADs were collected from the Shenzhen First-aid Command Center which receives calls from the whole city and covers most of the permanent population in Shenzhen. Therefore, the present results may be representative of the authentic association between temperature-related indices and EADs due to MBDs. Second, using CHAP data and bilinear interpolation method to estimate individual pollutant exposure can more accurately analyze the exposure effect of pollutants on mental health. Third, the lag distribution interaction model can quantitatively analyze the joint lag effect and marginal lag effect of NO_2_ and Humidex. However, some limitations should be addressed. First, the EADs data did not include detailed individual medical records, which may lead to inappropriate MBDs diagnoses. Our study is based on preliminary diagnosis data, specific subtypes of MBDs cannot be further analyzed. Moreover, our study had no access to information about personal lifestyle, socioeconomic status, occupation, education level, etc., so we could not correct the influence of these factors on the models and results. The following research will consider the above variables in the models, and the results will be more accurate.

## Conclusions

Our study indicated that NO_2_ was the primary pollutant in Shenzhen that affected the emergency ambulance dispatches due to mental and behavioral disorders, exhibiting significant immediate exposure effects and cumulative lag effects. As NO_2_ concentration increased, the risk of EADs due to MBDs showed a linear upward trend without a threshold. Our research also proved that the interaction between NO_2_ and Humidex would have an impact on EADs due to MBDs. Under specific lag durations, the synergistic interaction between NO_2_ and Humidex increased the risk of EADs due to MBDs, particularly when both NO_2_ and Humidex reached extreme values and it was reflected in both early and late lag periods. In terms of early lag periods, Humidex would interact with the long-term lag of NO_2_ at lag 0 and lag 1. Regarding late lag periods, Humidex would interact with the long-term lag of NO_2_. These findings suggested that Shenzhen residents should pay particular attention to the impacts of NO_2_ and Humidex on mental health in their daily lives. Health education and promotion activities targeting NO_2_ and Humidex threshold values and vulnerable populations should be implemented by the healthcare authorities in Shenzhen to enhance public awareness of prevention, ultimately reducing the burden of mental illness and emergency admissions in the city.

## Electronic supplementary material

Below is the link to the electronic supplementary material.

Supplementary Material 1

## Data Availability

All data generated or analysed during this study are included in this article. Further enquiries can be directed to the corresponding author.

## Abbreviations

CHAP: ChinaHighAirPollutants
CI: Confidence interval
DALYs: Disability-adjusted life years
EADs: Emergency ambulance dispatches
ICD-10: International Classification of Diseases
IQR: Interquartile range
MBDs: Mental and behavioral disorders
NO_2_: Nitrogen dioxide
OR: Odds ratio
