# Supplementary material for: The impact of air pollutants on emergency ambulance dispatches due to mental and behavioral disorders in Shenzhen, China
Source: BMC Public Health. 2025 Feb 18;25:673. doi: 10.1186/s12889-025-21781-w (PMC11837661; doi:10.1186/s12889-025-21781-w)
Supplement: Supplementary file 1 — Supplementary Material 1 [file 12889_2025_21781_MOESM1_ESM.docx]

**Supplementary material**

**The impact of air pollutants on emergency ambulance dispatches due to mental and behavioral disorders in Shenzhen, China**

Yuchen Tian, Ziming Yin, Peng Wang, Lei Li, Suli Huang, Jinquan Cheng, Hongwei Jiang, Ping Yin

**List of Tables and Figures**

**Table S1.** Summary of meteorological factors used in this study.

**Table S2.** The results of sensitivity analyses at lag 0–6.

**Table S3.** Specific odds ratios (95%CI) and corresponding *P* values that are statistically significant in distributed lag interaction model.

**Fig. S1.** The geographical location of the study area in China.

**Fig. S2.** Spearman’s correlation coefficients among different air pollutants and meteorological factors in Shenzhen, 2013–2020.

**Fig. S3.** Time-series plots for daily air pollutants, meteorological factors, and EADs due to MBDs in Shenzhen, 2013–2020.

**Fig. S4.** Exposure-response curves between NO_2_ exposure and EADs due to MBDs stratified by sex and age at lag 0–6.

**Fig. S5.** Sensitivity analysis when eliminating the data after the COVID-19 epidemic (lag 0–6).

**Table S1.** Summary of meteorological factors used in this study.

| Abbreviation | Names of indices | Definition/equation |
| --- | --- | --- |
| Raw meteorological indices | | |
| Tmean | daily mean temperature | Daily 24-hour average |
| RH | relative humidity | Daily 24-hour average |
| Composite meteorological variable | | |
| Humidex | Humidity index |  |

Abbreviations: Tmean, daily mean temperature(℃); RH, relative humidity(%); Humidex, Humidity index.

**Table S2.** The results of sensitivity analyses at lag 0–6.

| Sensitivity analyses | Odds ratio (95%CI) | *χ²* value | *P* value |
| --- | --- | --- | --- |
| Single-pollutant model | | | |
| NO_2_ | **1.078(1.037,1.122)** | Ref |  |
| Two-pollutant model | | | |
| NO_2_+PM_2.5_ | **1.117(1.065,1.173)** | 6.25 | 0.012* |
| NO_2_+PM_10_ | **1.112(1.056,1.171)** | 3.25 | 0.071 |
| NO_2_+SO_2_ | **1.094(1.043,1.147)** | 1.18 | 0.277 |
| NO_2_+O_3_ | **1.083(1.040,1.128)** | 0.96 | 0.327 |
| NO_2_+CO | **1.100(1.055,1.148)** | 7.28 | 0.007* |
| Excluding the data post the COVID-19 epidemic | | | |
| NO_2_ | **1.067(1.023,1.112)** | — | — |

Abbreviations: PM_2.5_, particulate matter less than 2.5mm in aerodynamic diameter; PM_10_, particulate matter less than 10mm in aerodynamic diameter; NO_2_, nitrogen dioxide; SO_2_, sulfur dioxide; O_3_, ozone; CO, carbon monoxide; Statistical significance is shown in bold.

**Table S3.** Specific relative risk (95%CI) and corresponding *P* values that are statistically significant in distributed lag interaction model.

| Interaction items | Odds ratio (95%CI) | *P* value |
| --- | --- | --- |
| NO_2_ lag 0 : Humidex lag 0 | **1.001(1.000,1.001)** | 0.016* |
| NO_2_ lag 1 : Humidex lag 1 | **1.002(1.001,1.003)** | 0.002* |
| NO_2_ lag 1 : Humidex lag 8 | **1.001(1.000,1.002)** | 0.030* |
| NO_2_ lag 9 : Humidex lag 3 | **1.001(1.000,1.002)** | 0.048* |
| NO_2_ lag 12 : Humidex lag 2 | **1.001(1.000,1.003)** | 0.014* |
| NO_2_ lag 12 : Humidex lag 7 | **1.001(1.000,1.002)** | 0.025* |
| NO_2_ lag 13 : Humidex lag 1 | **1.001(1.000,1.002)** | 0.033* |
| NO_2_ lag 13 : Humidex lag 3 | **1.001(1.000,1.003)** | 0.014* |
| NO_2_ lag 13 : Humidex lag 8 | **1.002(1.000,1.003)** | 0.005* |
| NO_2_ lag 14 : Humidex lag 9 | **1.001(1.000,1.002)** | 0.034* |

Abbreviations: NO_2_, nitrogen dioxide; Humidex, Humidity index; Statistical significance is shown in bold; * means *P*<0.05.


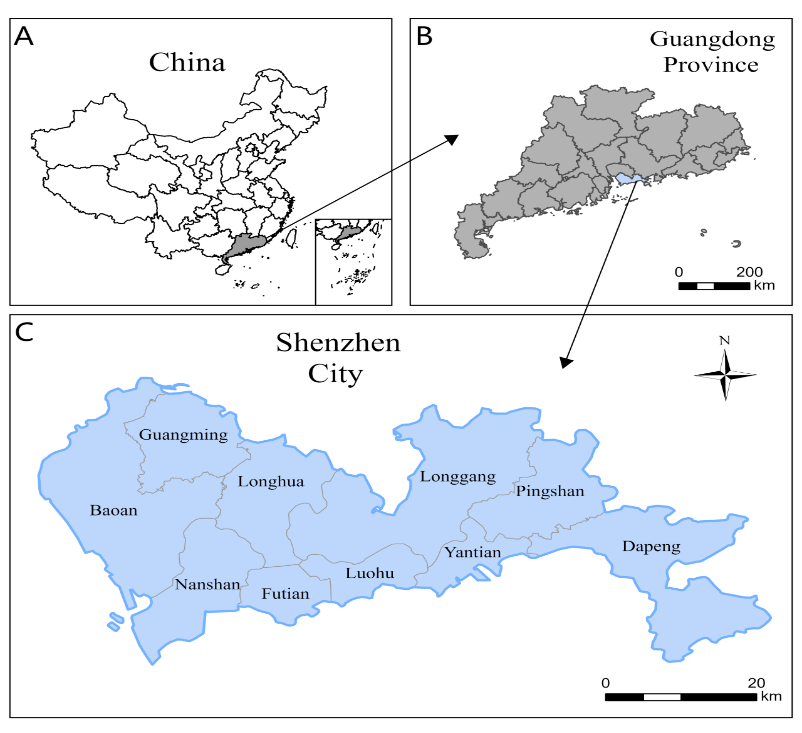


**Fig. S1.** The geographical location of the study area in China. (A) China;(B) Guangdong Province; (C)Shenzhen City.


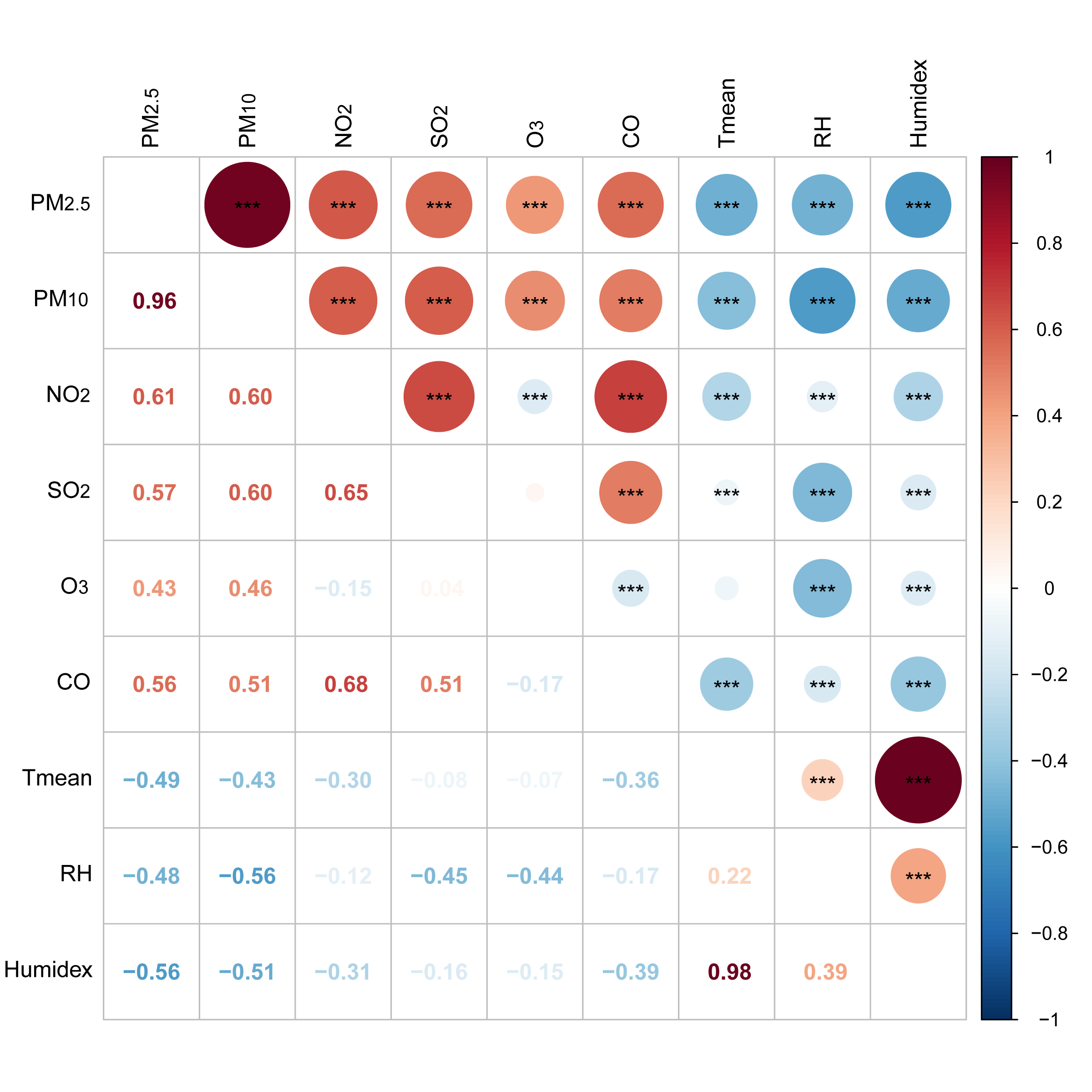


**Fig. S2.** Spearman’s correlation coefficients among different air pollutants and meteorological factors in Shenzhen, 2013–2020.

Abbreviations: PM_2.5_, particulate matter less than 2.5mm in aerodynamic diameter; PM_10_, particulate matter less than 10mm in aerodynamic diameter; NO_2_, nitrogen dioxide; SO_2_, sulfur dioxide; O_3_, ozone; CO, carbon monoxide; Tmean, daily mean temperature; RH, relative humidity; Humidex, Humidity index.

* means *P*<0.05, ** means *P*<0.01, *** means *P*<0.001.


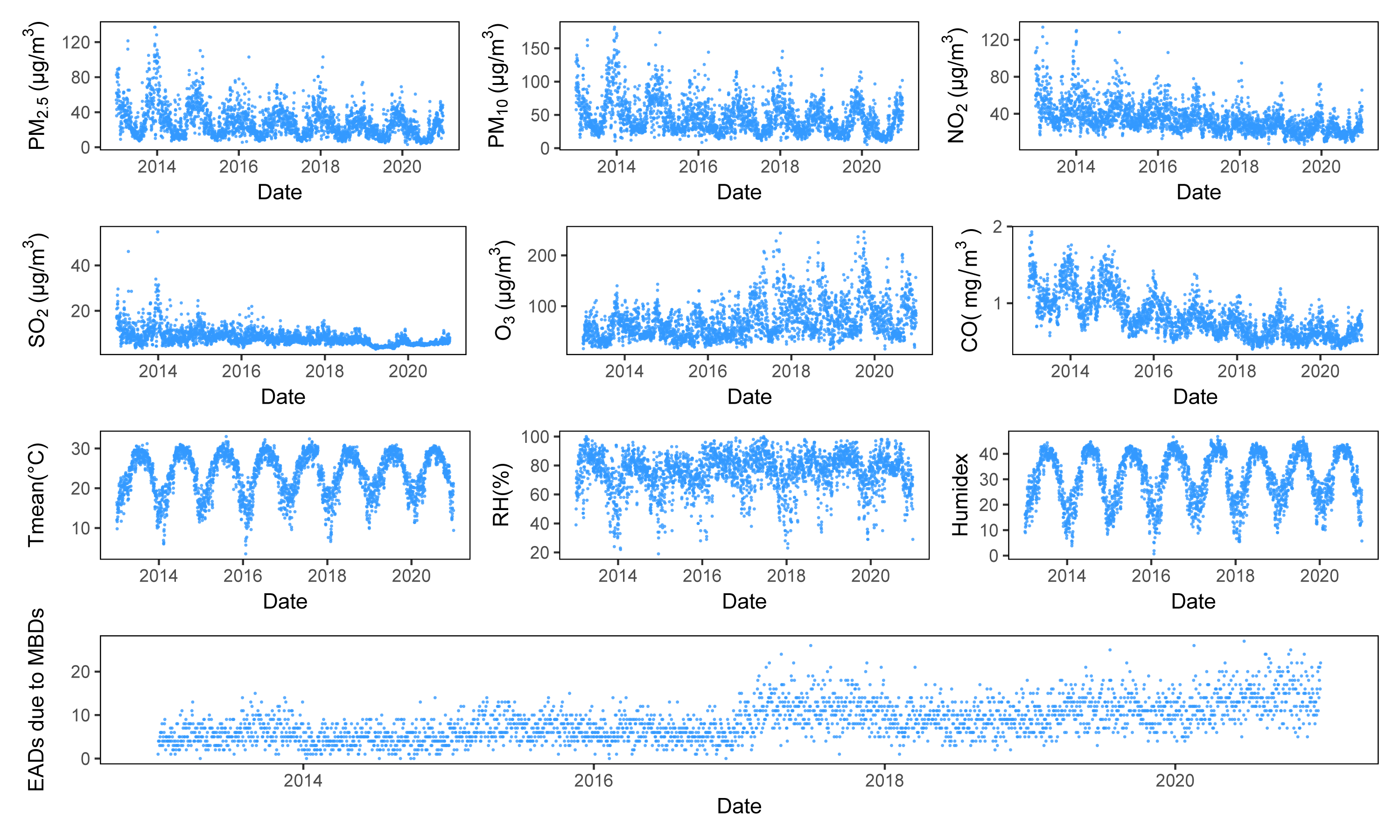


**Fig. S3.** Time-series plots for daily air pollutants, meteorological factors, and EADs due to MBDs in Shenzhen, 2013–2020.

Abbreviations: PM_2.5_, particulate matter less than 2.5mm in aerodynamic diameter; PM_10_, particulate matter less than 10mm in aerodynamic diameter; NO_2_, nitrogen dioxide; SO_2_, sulfur dioxide; O_3_, ozone; CO, carbon monoxide; Tmean, daily mean temperature; RH, relative humidity; Humidex, Humidity index; EADs due to MBDs, emergency ambulance dispatches due to mental and behavioral disorders.


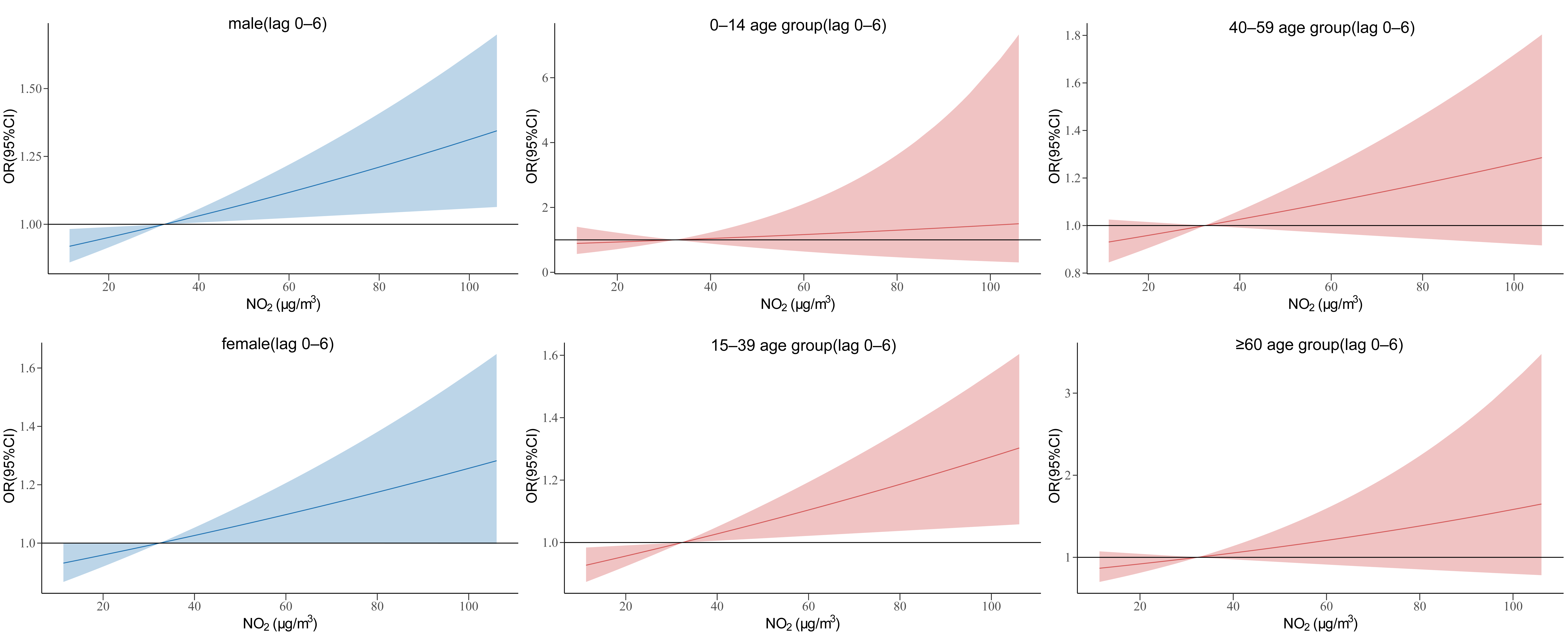


**Fig. S4.** Exposure-response curves between NO_2_ exposure and EADs due to MBDs stratified by sex and age at lag 0–6.

Abbreviations: EADs due to MBDs, emergency ambulance dispatches due to mental and behavioral disorders; NO_2_, nitrogen dioxide.


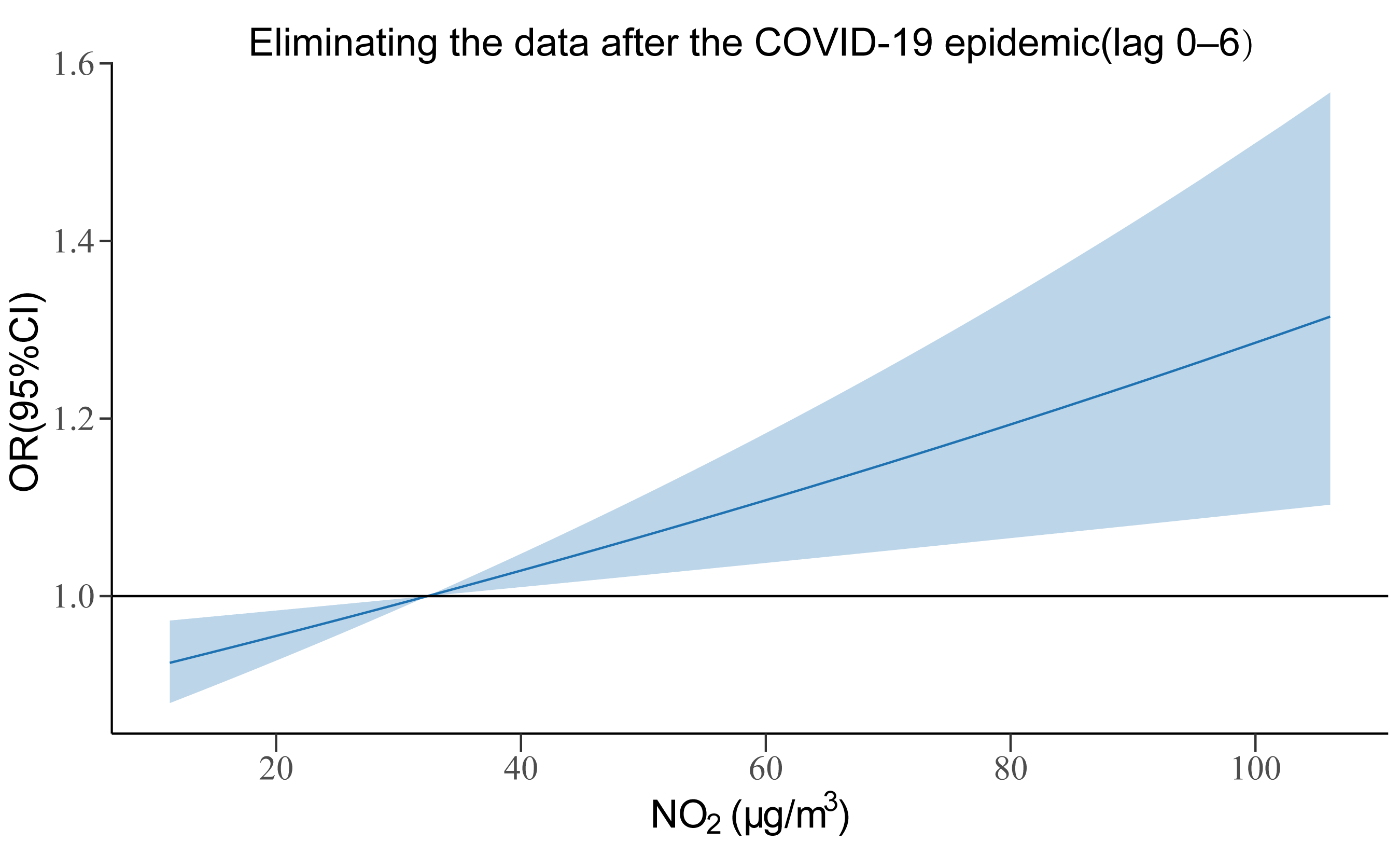


**Fig. S5.** Sensitivity analysis when eliminating the data after the COVID-19 epidemic (lag 0–6).
